# The role of Micro-RNAs in Hepatocellular Carcinoma: From Molecular Biology to Treatment

**DOI:** 10.3390/molecules19056393

**Published:** 2014-05-19

**Authors:** Marco D’Anzeo, Luca Faloppi, Mario Scartozzi, Riccardo Giampieri, Maristella Bianconi, Michela Del Prete, Nicola Silvestris, Stefano Cascinu

**Affiliations:** 1 Department of Medical Oncology, Università Politecnica delle Marche, A.O.U. “Ospedali Riuniti” Via Conca 71, 60126 Ancona, Italy; E-Mails: m.danzeo@gmail.com (M.D.); lucafaloppi@gmail.com (L.F.); kakeru@libero.it (R.G.); stellabianconi@gmail.com (M.B.) micheladelprete@gmail.com (M.D.P.); cascinu@yahoo.com (S.C.); 2 Istituto Oncologico “Giovanni Paolo II”, I.R.C.S.S. Ospedale Oncologico di Bari, Viale Orazio Flacco 65, 70124 Bari, Italy; E-Mail: n.silvestris@oncologico.bari.it

**Keywords:** HCC, hepatocellular carcinoma, miRNAs

## Abstract

Hepatocellular carcinoma (HCC) is the fifth most common cancer worldwide and the third leading cause of cancer deaths. microRNAs (miRNAs) are evolutionary conserved small non-coding RNA that negatively regulate gene expression and protein translation. Recent evidences have shown that they are involved in many biological processes, from development and cell-cycle regulation to apoptosis. miRNAs can behave as tumor suppressor or promoter of oncogenesis depending on the cellular function of their targets. Moreover, they are frequently dysregulated in HCC. In this review we summarize the latest findings of miRNAs regulation in HCC and their role as potentially diagnostic and prognostic biomarkers for HCC. We highlight development of miRNAs as potential therapeutic targets for HCC.

## 1. Introduction

Hepatocellular carcinoma (HCC) is the most common cancer of the liver, the fifth most frequently diagnosed cancer and the third leading cause of cancer-related deaths in the World, with an annual mortality of about 700,000 persons [1]. An estimated 80%–90% of HCCs develop in cirrhotic liver as a consequence of chronic viral hepatitis B (HBV) or C (HCV) [2]. HCC has a very low overall 5-year survival rate, ranging from 0% to 14% from the time of clinical diagnosis because it is difficult to diagnose HCC in its early stages [3,4]. Moreover, resistance to treatment, tumor recurrence or progression, and metastasis call for novel sensitive and specific molecular biomarkers for early diagnosis, to predict prognosis and to develop more effective therapeutic strategies able to improve the clinical outcome of HCC.

MicroRNAs (miRNAs) are a class of short (20–25 nucleotides in lenght) non-coding RNAs. To date, there are 1,870 human miRNA sequences registered in the miRBase database [5]. miRNA control the stability and translation of targeted messenger RNAs (mRNAs) through complementary interaction with the 3' untranslated region of target genes (or of complementary sequences). They are estimated to regulate expression of about one-third of human genes [6,7]; one miRNA can interact with hundreds of genes. Therefore, it will not be surprising that even a single type of miRNA may enable normal cells to be transformed into cancerous cells [8,9,10,11,12]. miRNAs indeed are involved in fundamental cellular processes, like embryonic development, differentiation, cell cycle, metabolism, and in carcinogenesis and tumor progression [13,14,15,16,17]. Here, we review miRNAs biogenesis, their alterations and their role in the diagnosis and treatment of HCC.

## 2. miRNAs Biogenesis

miRNAs are transcribed by the polymerase II as primary miRNA (pri-miRNAs); these transcripts are processed in the nucleus by ribonuclease III Drosha in combination with its cofactor, DGCR8 (DiGeorge syndrome critical region gene 8) into a precursor miRNA (pre-miRNA). This 70-90-nucleotide hairpin-shaped pre-miRNA is exported to the cytoplasm by exportin-5 and is further cleaved by another nuclease, Dicer, and its partner, HIV-1 transactivating response RNA binding protein (TRBP), to form an 18–25 nucleotides miRNA duplex [18,19]. While the passenger miRNA strand is typically degradated, the guide strand is incorporated into the RNA-induced silencing complex (RISC) and then mediate mRNA degradation or translational inhibition, based on the level of complementary base pairing between the miRNA and the 3' untranslated region (UTR) of target mRNA [20].

## 3. miRNAs and HCC

miRNAs are characterized by key regulatory functions and regulate many biological processes; alterations in their expression contribute to cancer. In fact, the involvement of miRNAs in tumorigenesis and tumor progression is well established, as they can behave as tumor suppressor or promoter of oncogenesis depending on the cellular function of their targets [21].

In particular, various miRNAs were deregulated (or are aberrantly expressed) in human HCC (Table 1) [22]. In most cases, HCC originates on a background of cirrhosis, a chronic and diffuse hepatic disease that results from continuous liver injury and regeneration, due to different etiological factors. Different etiologies of HCC play a role in the different miRNA expression profiles.

**Table 1:** miRNAs deregulated in hepatocellular carcinoma (HCC).

miRNAs	Molecularalteration	Targets	Characteristics	
miR-10a	Upregulated	EphA4, CADM1	EMT, metastasis
miR-17-5p	Upregulated	p38 pathway	Multiple tumor nodules, vein invasion, shortened overall survival
miR-18a	Upregulated	ER1a	Poor prognosis, poor differentiation, proliferation
miR-18b	Upregulated	TIMP3	Cell growth; tumorigenesis; metastasis
miR-21	Upregulated	C/EBPb, RhoB, PDCD4, PTEN	Drugresistance, metastasis
miR-23a	Upregulated	PGC-1a,G6PC	Gluconeogenesis
miR-130a	Upregulated	RUNX3	Drugresistance
miR-135a	Upregulated	FOXM1, MTSS1	Metastasis
miR-143	Upregulated	FNDC3B	Metastasis
miR-155	Upregulated	SOCS1, DKK1, APC, PTEN	High recurrence and poor prognosis following OLT, proliferation, tumorigenesis
miR-210	Upregulated	VMP1, AIFM3	Metastasis; apoptosis; proliferation
miR-216a	Upregulated	TSLC1	Tumorigenesis
miR-221	Upregulated	CDK inhibitors, p27, p57, Arnt, Bmf	Multinodularity, reduced time to recurrence, gain of metastatic properties, angiogenesis, apoptosis, proliferation, high tumor capsular infiltration
miR-224	Upregulated	NF-kB pathways, Atg5, Smad4, autophagy, API-5	Proliferation, apoptosis, metastasis
miR-301a	Upregulated	Gax	Metastasis
miR-373	Upregulated	PPP6C	Cell cycle
miR-490-3p	Upregulated	ERCIC3	EMT
miR-519d	Upregulated	CDKN1A/p21; PTEN; AKT3; TIMP2	Proliferation, invasion, apoptosis
miR-550a	Upregulated	CPEB4	Metastasis
miR-590-5p	Upregulated	TGF-beta RII	Metastasis, proliferation
miR-615-5p	Upregulated	IGF-II	Cell growth, migration
miR-657	Upregulated	TLE1, NF-jB	Proliferation
let-7 a,b,c,d,f,g	Downregulated	STAT3	Apoptosis, proliferation, earlyrecurrence
miR-1	Downregulated	ET1	Proliferation
miR-7a	Downregulated	PIK3CD, Caspase-3, HMGA2, C-myc, Bcl-xl	Proliferation, apoptosis, tumorigenesis, metastasis
miR-26a/b	Downregulated	IL-6, CyclinD2, E2	Poorsurvival
miR-34a	Downregulated	CCL22, c-met	Metastasis
miR-101	Downregulated	EZH2, EED, SOX-9, DNMT3A, Mcl1, Fos	Advanced tumor progression, poor prognosis, apoptosis; DNA methylation
miR-122	Downregulated	c-Myc, Bcl-w, ADAM-1, Wnt-1, MTTP, IL-6, TNF, IGF-1R, Cyclin G1	Gain of metastatic properties, early recurrence, angiogenesis, apoptosis
miR-124	Downregulated	ROCK2, EZH2, PIK3CA, CDK6, VIM, SMYD3, IQGAP1	Proliferation
miR-125a/125b	Downregulated	MMP11, SIRT7, VEGF-A, LIN28B2, Bcl-2, Mcl-1, Bcl-w	Angiogenesis, apoptosis, metastasis, proliferation[
miR-138	Downregulated	CCND3	Cell cycle
miR-139	Downregulated	ROCK2, c-fos	Metastasis
miR-145	Downregulated	IRS1, IRS2, IGF-1R, b-catenin, OCT4	Insulin-like growth factor pathway, stem-like cells tumorigenicity
miR-195	Downregulated	NF-jB pathway, VEGF, VAV2, CDC42, Cyclin D1, CDK6, E2F3	Proliferation, apoptosis, tumorigenicity
miR-199a/b-3p	Downregulated	PAK4, c-Met, mTOR, DDR1, caveolin-2	Reduced time to recurrence, poor overall survival and progression-free survival rates, proliferation, autophagy, metastasis
miR-200a	Downregulated	HDAC4	Proliferation, metastasis;
miR-203	Downregulated	ABCE1	Proliferation
miR-214	Downregulated	HDGF, catenin	Proliferation, angiogenesis, metastasis
miR-219-5p	Downregulated	GPC3	Proliferation
miR-223	Downregulated	STMN1	Predictor of overall survival and recurrence-free survival after LT
miR-375	Downregulated	ATG7, AEG-1	Autophagy
miR-376a	Downregulated	PIK3R1	Apoptosis, proliferation
miR-449	Downregulated	c-MET	Apoptosis, proliferation
miR-450a	Downregulated	DNMT3a	Proliferation
miR-520e	Downregulated	MEKK2; cyclin D1	Cell growth, proliferation

Cellular miRNAs, like miR-155 and miRNA-18a, seem able to regulate HBV infection at the transcription level either by targeting cellular transcription factors required for HBV gene expression or by a directly binding to HBV transcripts [23,24,25]. miRNAs can target important players in DNA methylation and histone modification that play crucial roles in HBV cccDNA transcription [26,27]. On the other hand, HBV encoded proteins can influence cellular miRNA expression [28].

HCV chronic infection is another independent etiological factor for HCC. miR-196 plays a protective role in HCV-induced HCC by upregulating hemeoxygenase 1 (HMOX1) expression and inhibiting HCV transcription [29].

Cirrhosis caused by chronic alcohol consumption is a risk factor of HCC especially in western countries. miR-217 could promote ethanol-induced fat accumulation in hepatocytes by downregulating SIRT1 [30] and miR-126 was found decreased in alcohol-related HCC [31]. In a preclinical model, mice with an ethanol-containing diet, miR-199 and miR-200, commonly downregulated in HCC, were found decreased.

miRNAs are also involved in the pathogenesis of Non Alcoholic Steato Hepatitis (NASH), an increasingly important risk factor for HCC in recent years.

Unsaturated fatty acids have been shown to increase miR-21 expression which downregulates the expression of tumor suppressor phosphatase and tensin homolog (PTEN) [32]. MiR-155, which targets another tumor suppressor C/EBP-𝛽 is consistently upregulated in choline-deficient and amino acid-defined (CDAA) fed mice [24].

Many studies have shown differences of miRNAs expression in HCC tissues compared to non-tumoral tissues. An important note is that these works are qualitatively heterogenic, as each study identify a unique profile. This can be due to various factors, first the variability in the technical procedure: studies have been performed using the three most common detection methods such as microarray, RT-qPCR and next-generation sequencing (NGS).

Hou *et al*. performed their study using NGS, which is a high-throughput technology that provides global information on all miRNAs in a sample [33]. They analyzed the miRNomes in human normal liver, hepatitis liver, and HCC. They found nine miRNAs accounted for ~88.2% of the miRNome in human liver. The three most represented miRNAs were mRr-122, miR-192, and miR 199 a/b-3p. In HCC miR-199a/b-3p is consistently decreased, this is associated with poor prognosis. Furthermore, both *in vitro* and *in vivo*, the PAK4/Raf/MEK/ERK pathway is inhibited by miR-199a/b-3p targeting tumour-promoting PAK4 to suppress HCC growth. In this study the expression of miR-122 decreased only in half of the HCCs and that is poorly relevant for survival. In contrast, there are many studies that described a tumourigenic role of miR-122 [8,34,35,36], including Gramantieri *et al*. who found that 35 miRNAs, including miR-122, differ between HCC and cirrhosis. A recent study performed with microarray, which is based on annealing of DNA oligonucleotides to the homologous sequences, on a microchip, identified 40 miRNAs with significantly different expression between normal and HCC cell lines. Hierarchical clustering analysis showed up-regulated hsa-miR-1308 and down-regulated hsa-miR-122 as the top two differently expressed miRNAs [37].

These studies associated loss of miR-122 expression in HCC with poor prognosis and metastasis. In fact, the liver-specific miR-122 is the most abundant miRNA in the liver, modulates hepatic lipid metabolism and it plays an important role in regulating hepatocyte development and differentiation [38,39]. Loss of its expression correlates with impairment of mitochondrial metabolism [40]. On the contrary, restoration of miR-122 reduces the invasion, migration, and growth of metastatic liver cancer cells [41]. It functions as a potential tumour suppressor in two ways: inhibiting hepatic cell growth by targeting cyclin G1 and promoting apoptosis of hepatic cells by targeting BCL-w and ADAM17 involved in metastasis [33,41,42]. Besides miR-122, there are many miRNAs, with upregulation (miR-21, miR 221, miR224) or down regulation (miR-223, miR-1, miR-101), that are involved in promoting loss of cell cycle control in HCC [8,11,43,44,45,46,47]. miR-21 promotes cell proliferation and tumor invasiveness in HCCs by targeting PTEN, PDCD4, and RECK [48]. miR-221 promotes the cell-cycle progression by suppressing the expression of cyclin-dependent kinase inhibitors CDKN1B/p27 [49] and CDKN1C/p57 [11]. Down regulation of miR-1 and miR-101 inhibits apoptosis of HCC cells and silencing miR-1 mediate HCC cell invasion [46,47]. Upregulation of miR-155 promote hepatocyte proliferation and tumor genesis by increasing Wnt signalling [23,50]. Instead, some miRNAs (miR-9, miR-143, miR-30d, and miR-151) have been shown to promote HCC metastasis [51,52].

Hence, miRNAs expression can be modified by mutations [53], polymorphisms (SNPs) [54,55], transcriptional deregulation, defects in the miRNAs biogenesis machinery [56], and epigenetic changes [57], such as DNA methylation that silences genes that encode miRNAs. Recently other miRNAs (miR-494, miR-429, miR-101) have shown their relevance in HCC [58,59,60].

## 4. miRNAs in HCC Diagnosis

Since the reliability of laboratory analysis biomarkers [α-fetoprotein (AFP) and des-γ-carboxy-prothrombin (DCP)] is still questionable, the accuracy of AFP is modest, especially in benign liver diseases, such hepatitis and cirrhosis and the elevated DCP activity is only ~50% of HCC patients with tumours <3 cm [61,62], novel biomarkers for early HCC diagnosis are urgently needed. miRNAs have been very promising as diagnostic markers of HCC. In fact, miRNAs are stable in human serum/plasma as free miRNAs released from cancer cells; many studies shown that circulating miRNAs are resistant to RNase activity and extreme pH and temperature [63,64]. Although a number of HCC-associated miRNAs sets have been identified, only few of them have been validated to assist in diagnosis of HCC. Li *et al*. identified a panel of 13 miRNAs that can serve for HBV-positive HCC diagnosis [65]. Moreover, three of these miRNAs (let-7f, miR-375, and miR-25) can discriminate HCC patients from healthy subjects. A recent study showed that a seven-miRNAs set (miR-21, miR-26a, miR-27a, miR-122, miR-192, miR-223, and miR-801) can differentiate HCC form healthy, chronic hepatitis B and cirrhosis groups and it is a promising biomarker for the early HCC diagnosis [66]. Interestingly, miRNAs could be powerful markers for classifying HCC subtypes. Based on the expression of AFP and EpCAM (epithelial cell adhesion molecule), Yamashita *et al*. classified HCC cells into EpCAM^+^ AFP^+^ with metastatic properties and poor prognosis, and EpCAM^-^ AFP^-^ cells with good outcome [67,68]. Later, a 20-miRNAs panel has been identified to distinguish HpSC-HCC (hepatic stem cell-like hepatocellular carcinoma) from MH-HCC (mature hepatocyte--like hepatocellular carcinoma) [69]. These results could significantly predict the two HCC subtypes with an overall accuracy of 78% and demonstrated that specific miRNAs are preferentially expressed in HpSC-HCC.

All of these studies suggest that miRNAs may be useful as novel biomarkers in the early and non-invasive detection of HCC.

## 5. miRNAs in HCC Prognosis

Although current markers used for HCC prognosis are still based on clinical parameters, biomarkers related to metastasis and disease recurrence might be used as prognostic factors. For example, to predict metastatic potential and tumor recurrence Budhu *et al*. identified a 20-miRNAs signature able to classify HCC patients, in every stage [10]. Moreover, it was an indipendent and significant predictor of patient prognosis, who would benefit from adjuvant therapy to reduce the chance of metastasis and disease recurrence. 

Wong *et al*. revealed that miRNAs expression profiles of primary HCC and venous metastasis are similar [70]. These data suggest that early miRNAs deregulation prompts later metastatic development of primary HCC. 

In a large cohort of patients Ji *et al*. showed the importance of miR-26 in determining the outcome of HCC patients [71]. Especially tumors with reduced miR-26 expression had a distinct gene-expression profile, and patients whose tumors had low miR-26 expression, compared with patients whose tumors had high miR-26 expression, presented a shorter survival but a better response to interferon alfa treatment.

Recently, Wei *et al*. identified a novel 20-miRNAs signature that can predict the survival of patients with hepatocellular carcinoma [72]. This provides a potentially valuable approach for evaluating the prognosis of patients with hepatocellular carcinoma and helping clinicians to design appropriate treatment plans.

## 6. miRNAs in HCC Treatment

Recent evidence has suggested the potential application of miRNAs as novel strategy in cancer therapy for HCC. In fact, the discovery of miRNAs as an important player in the development and progression of HCC has implied that miRNAs can be used as therapeutic agents. 

The complexity of miRNAs biology offers a novel mechanism of action for therapeutic intervention but also poses unique challenges for the development of therapeutic modulators as drugs. The therapeutic application of miRNAs involves two strategies. 

The first aims to inhibit oncogenic miRNAs by using miRNA antagonists, such as antimiRs, locked nucleic acids (LNAs) or antagomiRs [73]. MicroRNA antagonists are single-stranded RNA molecules, approximately 21–23 nt long, that act through complementary base-pairing with miRNAs. To achieve effective pharmacological inhibition of disease-associated miRNAs, miRNA antagonists contain chemical modifications to enhance binding affinity, confer nuclease resistance, and facilitate cellular uptake [73].

The second strategy, miRNA replacement, involves the reintroduction of a tumor suppressor miRNA mimetic to restore a loss of function [74]. miRNA mimetics represent an additional level of complexity compared with antimiRs. This strategy could have the potential to induce unwanted effects when novel miRNAs are introduced into a cell, but currently *in vivo* evidence for toxicity induced by miRNA mimetics is still lacking.

As discussed earlier, because one miRNA can target multiple genes and pathways, artificially increasing or decreasing the expression level of a given miRNA could enhance the biological efficacy and reduce the risk of resistance to therapy. But, for the same reason, the modulation of miRNAs expression could also result in undesiderable off-target effects. 

Another problem of miRNAs-based anticancer therapies is their delivery. One opportunity is represented by the adeno-associated viral vectors thanks to the lower risk of vector-related toxicities and good gene transfer efficacy. Kota *et al*. administered systemically mir-26a (its function as tumor suppressor by suppressing the G_1_/S transition and is frequently downregulated in HCC) in a mouse model using adeno-associated virus and found that ectopic expression of miR-26a results on inhibiton of cancer cell proliferation, induction of tumour-specific apoptosis [75], suggesting the therapeutic potential of restoring the expression of a dysregulated miRNAs to treat HCC. Moreover, this work suggests that delivery of miRNAs may provide an important therapeutic strategy. However, its significance in patients still need to be confirmed. 

Some works identified a correlation between modulation of cellular miRNAs abundance and the sensitivity of HCC cancer cells to chemotherapeutic drugs. Loss of miR-122 facilitates its tumourigenic role such as cell migration and survival, while overexpression and restoration of miR-122 increases the sensitivity of HCC cells to chemotherapeutic agents, such as doxorubicin or sorafenib, a multi-kinase inhibitor targeting both Raf and RTKs as well as PDGFR [76,77,78].

From a more clinical point of view, a phase I trial investigating the role of cancer-targeted miRNA drug MRX34, a liposome-based miR-34 mimic, is currently undergoing [79]. Further larger trials are needed to assess the role of miRNA based drugs in clinical practice [80].

## 7. Conclusions

miRNAs, small non-coding RNAs that negatively control gene expression, are important players in many cellular processes, including carcinogenesis. Aberrantly expressed miRNAs in HCC, associated with the identification of miRNA signatures, which are correlated with HCC progression or metastasis, make them potential diagnostic and/or prognostic markers and provide novel therapeutic targets for HCC treatment. miRNA signatures need to be further validated with high accuracy in future studies with larger samples, avoiding variations in the technical procedures, from the method of sampling to the method of detection. Furthermore, miRNA-based gene therapy offers a great challenge to improve diagnosis and treatment of HCC.
